# Influence of the Medial and Lateral Posterior Tibial Slope on Anterior Tibial Translation During Gait in Anterior Cruciate Ligament–Deficient Knees

**DOI:** 10.1177/23259671251387352

**Published:** 2025-12-09

**Authors:** Moses K.D. El Kayali, Philippe Moewis, Benjamin Bartek, Sven Scheffler, Heide Boeth, Tobias Winkler, Georg N. Duda, Tobias M. Jung, Stephan Oehme

**Affiliations:** * Charité–Universitätsmedizin Berlin, corporate member of Freie Universität Berlin, Humboldt-Universität zu Berlin, Center for Musculoskeletal Surgery, Berlin, Germany; † Berlin Institute of Health at Charité–Universitätsmedizin Berlin, Julius Wolff Institute, Berlin, Germany; ‡ Sporthopaedicum Berlin, Germany; § Berlin Institute of Health Center for Regenerative Therapies, Berlin Institute of Health at Charité–Universitätsmedizin Berlin, Berlin, Germany; Investigation performed at Charité–Universitätsmedizin Berlin, corporate member of Freie Universität Berlin, Humboldt-Universität zu Berlin, Center for Musculoskeletal Surgery, Berlin, Germany

**Keywords:** ACL reconstruction, anterior cruciate ligament (ACL), anterior tibial translation, functional gait analysis, posterior tibial slope

## Abstract

**Background:**

The posterior tibial slope (PTS) is a key determinant of knee biomechanics, with increased PTS values identified as a risk factor for anterior cruciate ligament (ACL) injuries and ACL graft failure. While the relationship between PTS and passive anterior tibial translation (ATT) is well-documented, its influence on dynamic ATT during gait remains unclear.

**Purpose:**

To evaluate the in vivo association between medial and lateral PTS (MPTS and LPTS, respectively), as well as lateral-medial PTS difference (ΔPTS), with dynamic and passive ATT in ACL-deficient knees compared with contralateral ACL-intact knees.

**Study Design:**

Descriptive laboratory study.

**Methods:**

A total of 13 patients with unilateral ACL tears undergoing ACL reconstruction at the authors’ orthopaedic sports medicine center were included in the analysis. Dynamic ATT was assessed using a motion capture–based gait analysis system during walking, while passive ATT was measured using a KT-1000 arthrometer. The contralateral ACL-sufficient knees of the participants were used as paired controls. PTS values (MPTS, LPTS, ΔPTS [LPTS minus MPTS]) were measured from computed tomography. Correlation analyses were performed to evaluate the relationships between PTS parameters and ATT during gait, as well as during passive assessment.

**Results:**

MPTS, LPTS, or ΔPTS values did not differ significantly between ACL-injured and healthy knees (*P* > .05). No significant correlations were identified in healthy knees or between MPTS/LPTS and ATT in either group.A strong inverse correlation was observed between ΔPTS and dynamic ATT in ACL-injured knees (*r* = −0.692; *P* = .014), particularly during the stance (*r* = −0.708; *P* = .015) and swing phases (*r* = −0.775; *P* = .005).

**Conclusion:**

The results suggest that lateral-medial asymmetry of the PTS (ΔPTS) may influence dynamic ATT in ACL-injured knees during gait. A greater LPTS relative to MPTS was associated with reduced dynamic ATT, whereas a lower LPTS relative to MPTS correlated with increased ATT. This directional relationship indicates that slope asymmetry could act as a restraint, potentially limiting anterior tibial shift during dynamic loading. The observed inverse correlation between ΔPTS and dynamic ATT indicates that this asymmetry could act as a restraint, potentially limiting anterior tibial shift. No significant correlations were identified between MPTS or LPTS individually and dynamic or passive ATT. Further studies are needed to validate these findings.

**Clinical Relevance:**

Considering the potential influence of lateral-medial tibial slope asymmetry on dynamic knee stability, these findings provide insight into the biomechanical function of the ACL-injured knee during gait.

Anterior cruciate ligament (ACL) injuries are among the most common knee injuries with an estimated annual incidence of 68.6 per 100,000 person-years.^35^ Resulting ACL deficiency can significantly affect knee stability, subjective knee function, and long-term joint health, often necessitating surgical reconstruction to restore biomechanical integrity.

The posterior tibial slope (PTS) is a crucial factor regarding knee stability, as it has a strong linear relationship to the amount of graft force experienced by the ACL in axially loaded knees.^3^ An increased PTS was identified as a risk factor for an ACL injury and for ACL graft failure.^15,28^ Furthermore, an increased medial PTS (MPTS) and an increased lateral PTS (LPTS) were found to be independent risk factors for an ACL injury.^16^

While the correlation between PTS and both passive anterior tibial translation (ATT) and ACL forces is well studied, its influence on dynamic ATT during gait remains less understood. Passive assessments eliminate the effects of muscle activation and joint loading, failing to capture the complexities of in vivo motion. In contrast, dynamic ATT during gait integrates muscle activation and neuromuscular compensations, making it clinically more relevant for understanding functional knee stability in ACL-deficient patients. Patients with an ACL deficiency showed even a decreased dynamic ATT during level walking compared with their healthy contralateral knee.^4^

Limited studies have investigated the influence of the PTS on gait biomechanics in ACL-deficient patients. A study using a computational model has indicated that an increase in PTS is associated with elevated tibial shear forces and greater ATT, with these effects being most pronounced during level walking.^29^ Another study, which simulated gait using a fine element model, showed similar results indicating that during the whole stance phase, an increase in PTS significantly increases ATT and ACL forces.^30^ Studies examining the effect of the PTS on knee biomechanics during gait using in vivo models are limited in the existing literature. A recent study by Zee etal^52^ identified no correlation between the PTS and dynamic ATT, emphasizing the need for more targeted research on how the PTS affects knee kinematics under dynamic conditions. The separate roles of the MPTS and LPTS on dynamic ATT during gait in ACL-deficient knees is even less understood.

Our study aims to address this gap in the literature by analyzing the influence of MPTS and LPTS on ATT during gait in ACL-deficient knees. To achieve this, we conducted an in vivo analysis of patients with ACL deficiency, combining a motion capture–based gait analysis to evaluate dynamic ATT with computed tomography (CT) imaging to accurately determine MPTS and LPTS. We hypothesized that MPTS, LPTS, and the difference between MPTS and LPTS (LPTS minus MPTS; ΔPTS) are significantly correlated with dynamic ATT during gait in ACL-deficient knees.

## Methods

### Patient Cohort

Patients with unilateral ACL tears (mean age, 35.77 [range, 24-49 years]; mean body mass index, 26.04 [range, 21.01-30.48]; 4 women and 9 men; mean time after injury, 3.83 months [range, 1-9 months]) were recruited into the study during routine clinical consultations at our orthopaedic sports medicine center. Patients aged >18 years with a unilateral ACL injury confirmed by physical examination and magnetic resonance imaging, an intact contralateral knee on physical examination, and scheduled for ACL reconstruction were screened for inclusion. All measurements were performed 1 to 13 days before ACL reconstruction. The contralateral ACL-sufficient knees of the participants were used as paired controls.^23^

Patients were excluded if they met any of the following criteria: a history of fracture, osteotomy, or previous ligament reconstruction surgery in the lower extremities or spine; neurological conditions associated with musculoskeletal disorders; or any other musculoskeletal pathology of the lower limb, such as concomitant ligament or meniscal injuries. The study was approved by the local ethics committee (Ethikkommission der Charité–Universitätsmedizin Berlin) and was performed in accordance with the ethical guidelines of the Declaration of Helsinki. All patients provided written informed consent before participation and were properly informed about the measurement procedures.

### Assessment of Anterior Tibial Translation During Dynamic Activities

**Figure 1.**
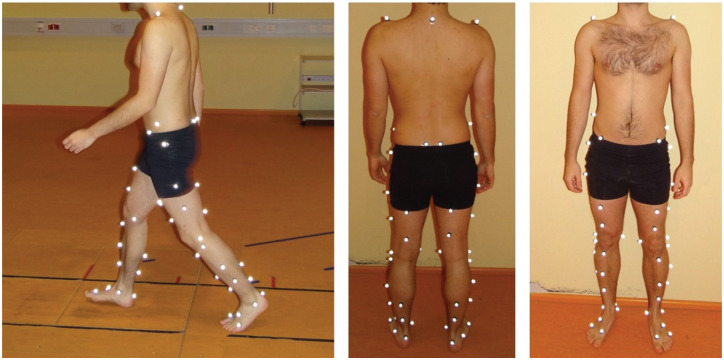
Representative images illustrating the marker set used in motion capture gait analysis to assess tibiofemoral translation during dynamic activities.

Knee joint kinematics during dynamic activity were assessed using a previously published and reliability tested optical motion capture system (Vicon) with 10 T20S infrared cameras recording at 120 Hz.^4,32^ A total of 52 reflective markers were placed on anatomic landmarks of the lower extremities according to a previously optimized configuration,^43^ and patients performed 3 to 6 walking trials at a self-selected pace (Figure 1).

To account for soft tissue artifact and define anatomic coordinate systems, the Ohio Scholastic Soccer Coaches Association method (a combination of optimum common shape technique [OCST], symmetrical axis of rotation analysis [SARA], and symmetrical center of rotation estimation [SCoRE] techniques) was applied.^6,10,11,42,43^ Functional movements (eg, star-act motion, flexion-extension) were used to identify joint centers and axes of rotation. OCST was used to optimize marker clusters, and SCoRE and SARA were used to define the hip center and knee flexion axis, respectively. Tibiofemoral translations and rotations were calculated by tracking the femoral axis center relative to the tibial coordinate system, minimizing anteroposterior (AP) translation errors.

### Data Analysis

The 3-dimensional tibiofemoral motion data collected during walking were segmented into individual gait cycles. For each kinematic variable, 101 discrete data points corresponding to 0% to 100% of the gait cycle (heel strike to heel strike) were extracted at 1% intervals using interpolation. The AP range during each gait cycle was calculated as the difference between the minimal and maximal AP movements of the coordinate systems relative to each other. To analyze group differences, the kinematic curves were averaged across trials for each individual patient and subsequently across all patients within each cohort.

### Assessment of Passive Anterior Tibial Translation

Passive ATT was measured using a KT-1000 arthrometer (MEDmetric Corp). Measurements were performed with participants in the supine position and the knee flexed to 30°. The tibia was stabilized to minimize external rotation. A standardized anterior force of 133 N was applied to the tibia, and the resulting ATT was recorded in millimeters. Three measurements were obtained, and the mean value was used for analysis.

### Measurement of PTS

#### CT Protocol

All patients underwent CT imaging of the analyzed knee in a supine position with the knee extended and lower limb rotated with the patella directed anteriorly (~20 cm proximal and distal of the joint line, resolution 0.5 × 0.5 mm, slice thickness, 1 mm) (Toshiba Aquilion Prime; Toshiba), including standardized reconstructions of 3 orthogonal planes (coronal, axial, sagittal).

**Figure 2.**
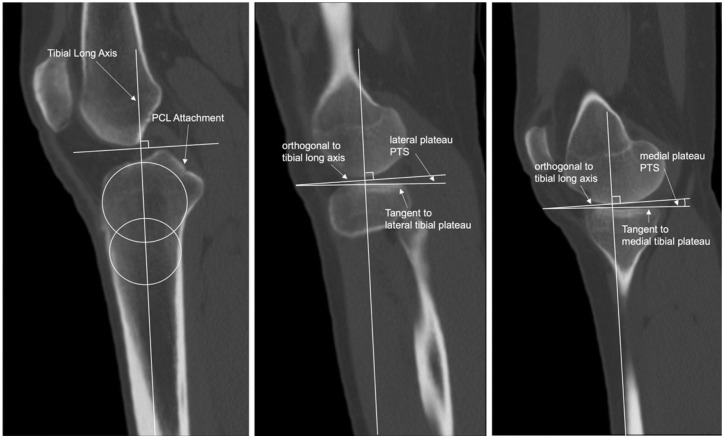
Representative images demonstrating the measurement of the posterior tibial slope (PTS) on computed tomography images in a left knee presented from left to right. Following the method by Hudek etal,^17^ the tibial long axis is identified on the central sagittal slice of the tibia (left image) and applied as an overlay on the image series. The PTS is measured by drawing a line along the superoanterior to superoposterior border of the subchondral bone at the coronal midpoint of the lateral tibial plateau (LTP) (central image) or medial tibial plateau (MTP) (right image). The angle between this line and a line perpendicular to the tibial long axis defines the PTS for the LTP and MTP. PCL, posterior cruciate ligament.

The PTS of the medial tibial plateau (MTP) and the lateral tibial plateau (LTP) were measured using a validated technique described by Hudek etal.^17^ The PTSdifferential (ΔPTS) was calculated as LPTS minus MPTS.^48^ The measurement technique is depicted in Figure 2.

Measurements were carried out using a picture archiving and communication system workstation (Centricity RIS-I 4.2 Plus; GE Healthcare).

For interreader reliability, measurements were conducted by 2 authors (S.O. and M.K.D.E.) blinded to each other’s measurements and to the kinematic assessment.

#### Statistical Analysis

A post hoc power analysis was carried out to calculate the statistical power achieved by the given sample size. Power for the dynamic ATT comparison was calculated to be 0.81 (paired *t* test, calculated effect size based on means and standard deviations: *d* = 0.93). For the passive ATT comparison, power was calculated to be 0.998 (paired *t* test, calculated effect size based on means and standard deviations: *d* = 1.48). Both analyses used an α error probability of .05 and were conducted using G*Power, Version 3.1.9.6.^12^

Descriptive parameters were analyzed, and the mean, standard deviation, and range were calculated where applicable. For quantitative variables, normality was assessed using the Shapiro-Wilk normality test. Paired *t*test was conducted when data were normally distributed; otherwise, the Wilcoxon signed-rank test was used. The calculated mean value for MPTS, LPTS, and ΔPTS between raters were used for analysis.

Comparisons of ATT ranges between healthy and injured knees were conducted, as well as comparison of MPTS, LPTS, and ΔPTS between these groups. To assess the effect of PTS on ATT, the Pearson correlation coefficient was calculated (or the Spearman correlation coefficient if normality was not confirmed) between ATTranges (healthy and injured) and MPTS, LPTS, and ΔPTS.

To evaluate whether the effect of PTS on ATT differed between the stance and the swing phases of gait, the ATT range was calculated separately for each phase. The stance phase of gait was defined as the period between initial contact (vertical ground-reaction force [vGRF] > 20N) and toe-off (vGFR < 20N).^5^ ATT ranges of both phases were compared within healthy and injured knees, as well as between healthy and injured knees.

To evaluate the relationship between PTS and ATT, the Pearson correlation coefficient was calculated (after normality was confirmed) between ATT ranges (stance phase and swing phase) and MPTS, LPTS, and ΔPTS.

A Pearson correlation coefficient (*r*) of 0.0 to 0.19 was regarded as very weak, 0.2 to 0.39 as weak, 0.40 to 0.59 as moderate, 0.6 to 0.79 as strong, and 0.8 to 1.0 as very strong correlation.^37^ Interrater variability was assessed by calculating the intraclass correlation coefficient (ICC), which was categorized as slight (0.0-0.2), fair (0.21-0.4), moderate (0.41-0.6), good (0.61-0.8) or excellent (>0.8).^24^

For each correlation, the *r* and *P* values were reported. A 2-sided significance level of *P* < .05 was considered statistically significant.

All calculations were performed using Microsoft Excel for macOS (Version 2024; Microsoft Corp) and SPSS (Version 29.0.0.0; IBM SPSS Statistics for macOS).

## Results

A total of 38 patients were diagnosed with ACL injury and screened for eligibility. Of these, 19 patients matched the inclusion criteria and were invited to participate in the study. Of the 19 eligible patients, 6 declined to participate or did not attend the motion analysis session. Thus, 13 patients provided informed consent and were included in the study.

Measurements of both MPTS and LPTS demonstrated excellent interrater agreement between observers (MPTS: ICC, 0.983; LPTS: ICC, 0.969).

Dynamic ATT was significantly lower in ACL-injured knees (16.17 ± 2.18 mm) compared with contralateral healthy knees (18.46 ± 2.73 mm) (*P* = .013). Conversely, passive ATT was significantly higher in ACL-injured knees (8.78 ± 3.59 mm) than in contralateral healthy knees (4.38 ± 2.19 mm) (*P* < .001).

There were no significant differences in MPTS (ACL-injured, 6.68°± 3.76°; healthy, 6.93°± 3.63°; *P* > .05), LPTS (ACL-injured, 7.72°± 3.29°; healthy, 8.39°± 3.24°; *P* > .05), or ΔPTS (ACL-injured, 2.71°± 1.74°; healthy, 2.91°± 2.21°; *P* > .05) between ACL-injured and contralateral healthy knees.

There was a high, inverse, statistically significant correlation between ΔPTS and dynamic ATT in the ACL-injured group (*r* = −0.692; *P* = .014). As seen in Table 1, there was no statistically significant correlation between MPTS, LPTS, or ΔPTS and dynamic or passive ATT in other analyses.

**Table 1:** Spearman Correlation Coefficient and Significance Level for Correlation Between Dynamic/Passive ATT and Different PTS*^*a*^*

	*r* (*P*)	
	ACL Intact	ACL Injured
Dynamic ATT and medial PTS		
Overall	0.336 (.31)	−0.032 (.92)
Stance phase	−0.046 (.91)	0.086 (.80)
Swing phase	−0.028 (.94)	−0.087 (.80)
Dynamic ATT and lateral PTS		
Overall	0.156 (.65)	−0.146 (.64)
Stance phase	0.169 (.66)	−0.221 (.52)
Swing phase	−0.22 (.57)	−0.344 (.33)
Dynamic ATT and ΔPTS		
Overall	0.266 (.43)	−0.692 (.014*^*b*^*)
Stance phase	0.475 (.07)	−0.708 (.015*^*b*^*)
Swing phase	0.423 (.26)	−0.775 (.005*^*b*^*)
Passive ATT and medial PTS		
Overall	0.387 (.27)	0.429 (.19)
Passive ATT and lateral PTS		
Overall	0.042 (.91)	0.187 (.58)
Passive ATT and ΔPTS		
Overall	0.273 (.45)	−0.468 (.15)

a ACL, anterior cruciate ligament; ATT, anterior tibial translation; PTS, posterior tibial slope.

b Statistically significant at *P* < .05.

The dynamic ATT range during the stance phase was significantly lower compared with the swing phase in both healthy knees (10.45 ± 3.69 mm vs 17.36 ± 2.91 mm) and injured knees (9.06 ± 3.15 mm vs 15.79 ± 2.13 mm).

There was no significant difference in ATT range between healthy and injured knees during the stance phase (*P* > .05); however, a significant difference was observed in the swing phase (*P* = .04).

Pearson correlation analysis revealed a strong, significant inverse correlation between ΔPTS and ATT range both during the stance phase (*r* = −0.708; *P* = .015) and the swing phase (*r* = −0.775; *P* = .005) in the ACL-injured knees. As presented in Table 1, in healthy knees, no statistically significant correlation was found.

## Discussion

The primary aim of this study was to investigate the relationship between the PTS and ATT in ACL-injured and the contralateral healthy knees. Our key finding is that the ΔPTS is significantly associated with altered knee biomechanics under dynamic conditions in ACL-injured knees. Specifically, a greater LPTS compared with MPTS (ie, a positive ΔPTS) demonstrates a strong, inverse, and statistically significant correlation with dynamic ATT in ACL-injured knees, both during the swing and the stance phase of gait. Conversely, when the LPTS is equal to or smaller than the MPTS (ie, ΔPTS is zero or negative), dynamic ATT values are higher according to our data. However, this effect was not reproduced in the passive context in either ACL-injured or healthy knees. Furthermore, no significant associations were observed with MPTS or LPTS individually and dynamic or passive ATT in ACL-injured and healthy knees.

Dynamic ATT was significantly lower in ACL-injured knees compared with contralateral healthy knees, which aligns with previous studies showing altered tibiofemoral kinematics after ACL injury.^4,38,52^ This reduction may reflect adaptive mechanisms such as modified gait patterns or compensatory muscular changes during dynamic activities.^2,4,18,22,33,39^ It is likely that muscle activity plays a central role in this process. Previous studies have shown that changes in muscle activation patterns can significantly influence joint kinematics. However, the underlying mechanism, particularly the complex interactions between passive and active stabilizers of the knee after ACL rupture are not yet fully understood.^18,27,39,46,47^

Conversely, passive ATT was significantly higher in ACL-injured knees, consistent with the increased laxity typically observed in ACL-deficient knees.^7-9,26,30,36,45,49^

The ATT range was significantly lower during the stance phase compared with the swing phase in both healthy and injured knees. This difference reflects the biomechanical demands of weightbearing during the stance phase, where stabilizing forces restrict ATT.^25,34,41^ Notably, the ATT range during the swing phase was significantly different between ACL-injured and healthy knees, potentially due to compensatory mechanisms or altered neuromuscular control after injury.^18,39^

Although high PTS values are an established risk factor for ACL injury and graft failure,^15,16,28^ no statistically significant differences in MPTS, LPTS, or ΔPTS were observed between ACL-injured and healthy knees in our study. This may be attributed to the relatively small sample size in our study, which could limit the detection of variation in PTS values.

The PTS is an important factor influencing knee biomechanics and stability, as higher PTS values are associated with increased graft forces experienced by the ACL under passive conditions.^1,3,14,44,50^ Passive ATT assessment eliminates the effect of muscle activation and joint loading, failing to capture the complexities of in vivo motion. In contrast, assessment of dynamic ATT during gait incorporates the combined effects of muscle activation, neuromuscular compensation, and joint loading, making it clinically more relevant.

Limited studies have investigated the effect of PTS on ATT in a dynamic context, and even fewer have done so in vivo. Using finite element models of the knee joint, Marouane etal^29,30^ demonstrated that increased PTS values are associated with an increase of dynamic ATT. Using a motion capture system, Zee etal^52^ examined the influence of MPTS, LPTS, and ΔPTS on dynamic ATT in 10 patients with ACL-injured knees and found no significant correlation. Interestingly, a nonsignificant moderate-to-strong negative correlation between ΔPTS and dynamic ATT during level walking as well as single-leg hop for distance was found at 1 year after ACL reconstruction.

Our results align with those of Zee etal^52^ regarding the lack of correlation between dynamic ATT and MPTS or LPTS. However, we observed a significant negative association between ΔPTS and dynamic ATT in ACL-injured knees. We hypothesize that a greater asymmetry between the MTP and LTP create an effect that limits anterior tibial shift, potentially due to uneven loading or wedging between the tibial and femoral articular surfaces. This could effectively act as a restraining factor, especially in ACL-injured knees where stabilizing structures are compromised. This is supported by the moderate, negative correlation of ΔPTS and passive ATT in ACL-injured knees found in our study (see Table 1).

While ΔPTS may serve as a restraint against anterior tibial shift, it could also limit tibiofemoral rotational movements, extending its influence beyond purely translational effects. Rotation occurs between the tibia and femur from full extension (0°) to approximately 20° of knee flexion, playing a key role in the screw-home movement.^13,31^ This mechanism is particularly relevant under active conditions and during the swing phase, when the knee is unloaded and approaching full extension.^19,21^ This aligns with our findings, where we observed a stronger negative correlation between ΔPTS and dynamic ATT in the unloaded phase (see Table 1).

In contrast, during passive conditions (such as KT-1000 measurements) where axial loading, muscle activation, and rotational movements are absent, the interaction with ΔPTS is limited. This may explain why the observed effects of ΔPTS on ATT were primarily detected under dynamic conditions. Future studies are needed to further investigate the effect of ΔPTS on rotational movements in both ACL-injured and ACL-intact knees under dynamic and passive conditions to better understand its role in knee stability.

Adding to the significance of ΔPTS, increased lateral-medial asymmetry of the PTS has been identified as a risk factor of lateral meniscal posterior tears concomitant with ACL injury.^51^ Furthermore, this increased ΔPTS has been associated with an increased preoperative pivot shift in patients with ACL injury.^20^ One study proposed a mechanism suggesting that axial loading forces could cause the lateral side of the femur to slide posteriorly off the steeper LTP, using the flatter MTP as a pivot point.^40^ While this dynamic could explain the enhanced pivot-shift phenomenon in knees with greater lateral-medial slope asymmetry, it contrasts with our findings, which demonstrate a negative correlation between ΔPTS and dynamic ATT during gait. This discrepancy may be attributed to the different biomechanical contexts, as pivot-shift movements do not occur during gait. Consequently, the mechanisms underlying knee kinematics during gait and pivot-shift testing differ significantly.

### Limitations

This study has several limitations that should be addressed. First, the sample size is relatively small, which may limit the generalizability and statistical power of the findings. Second, muscular forces were not measured in this study. The use of electromyography could provide valuable insights into the influence of potential muscular compensatory mechanisms on dynamic ATT. Furthermore, contralateral uninjured knees were used as normal control knees. Kozanek etal^23^ demonstrated that there were no statistically significant differences in the in vivo kinematics of the uninjured contralateral knee joint in patients with acute unilateral ACL deficiency and patients without knee injury. A potential benefit of using contralateral knees is the ability to directly compare both legs of the same individual, eliminating interpatient variability. Nevertheless, future studies should include comparisons with the kinematics of matched controls. Additionally, further studies with larger sample sizes and a wider range of PTS values are needed to better understand the relationship between PTS and dynamic ATT, as this topic remains underexplored. Finally, future research should also investigate the biomechanical implications of lateral-medial PTS asymmetry, which may play a critical role in AP and rotational stability of the knee.

## Conclusion

This study highlights the significance of lateral-medial asymmetry of the PTS in influencing dynamic ATT in ACL-injured knees. ΔPTS was inversely correlated with dynamic ATT during gait, indicating that knees with greater LPTS compared with MPTS (positive ΔPTS) exhibit lower dynamic ATT, while knees with equal or lower LPTS compared with MPTS (zero or negative ΔPTS) showed higher dynamic ATT values. This suggests that lateral-medial slope asymmetry may act as a restraint, limiting anterior tibial shift during gait. No significant associations were found between MPTS or LPTS individually and dynamic or passive ATT. Further research with larger cohorts and broader PTS variations is needed to confirm these findings and explore the role of ΔPTS in AP and rotational knee stability.
